# Context-sensitivity and context-productivity: notions of “practice” and “practicality” in ethnomethodology and conversation analysis

**DOI:** 10.3389/fsoc.2024.1221026

**Published:** 2024-07-24

**Authors:** Christian Meyer

**Affiliations:** University of Konstanz, Konstanz, Germany

**Keywords:** social theory, practice theory, ethnomethodology, conversation analysis, practice, practicality

## Abstract

The text reconstructs the concepts of practice and practicality used in ethnomethodology and conversation analysis and examines their internal similarities and differences as well as similarities and differences to other practice theories. After a description of the characteristics of practice theories, the ethnomethodological perspective on practice and practicality is presented. Then, the use of the terms in conversation analysis is examined. Ethnomethodology uses the notions of “practice” and “practicality” to outline a non-metaphysical theory of social order in which the sharedness of rules or meanings is not presupposed. “Practical” here means that social action, and social order more generally, are practically grounded as well as temporally and situationally constrained. The fact that practical action is fundamentally situated and can only be understood “from within” establishes an essentially indexical character of practical action. In conversation analysis, “practices” are viewed as “context-free” but “context-sensitive” components that constitute action and as such become the objects of investigation. While some have diagnosed a departure of conversation analysis from its ethnomethodological roots, I argue that “context-freeness” and “context-sensitivity” should be complemented by “context-productivity” by reference to Garfinkel’s interpretation of Aron Gurwitsch’s gestalt phenomenology in order to formulate a more encompassing concept of practice.

## Introduction

The noun “practice” and the adjective “practical” are frequently used in theoretical and empirical texts situated within the conceptual framework of ethnomethodology and conversation analysis (henceforth CA). However, the terms usually remain theoretically unexplained and their use is inconsistent. Given the ubiquity of the terms within ethnomethodology since the 1960s, ethnomethodology is sometimes counted among the sociological “practice theories” that have experienced a resurgence in the 21st century under new theoretical auspices (see, e.g., some of the chapters in Knorr-Cetina et al., 2001). In these papers, those variants of practice theory that distance themselves from Marx and turn instead to Wittgenstein, pragmatism, and phenomenology as theoretical resources have been seen in particular as possible cognates of conceptions of “practice” and “practicality” in ethnomethodology and CA (see, e.g., all other chapters in Knorr-Cetina et al., 2001). However, since the use of these terms in ethnomethodology and CA remains mostly unexplained and inconsistent, these texts have a rather programmatic status. In my text, in contrast, I will analyze key publications in ethnomethodology and CA in order to reconstruct salient aspects of the meaning of “practice” and “practicality.” In particular, I will suggest a possible reading of the terms in light of recent findings on the influence of Aron Gurwitsch’s gestalt phenomenology on Garfinkel, reconciling possible divergences between ethnomethodology and CA.

In what follows, I will first provide some brief introductory observations about the variety of uses of the terms “practice” and “practical” in ethnomethodology and CA (Section I). I will then present key arguments of philosophical and sociological conceptualizations of “practice” (Section II). Section II is necessarily compressed, as these conceptualizations are far too multi-layered and complex for a brief presentation, but it will fulfill its purpose to serve as a historical and systematic foil for the discussion of the specifics of the uses of the terms in ethnomethodology and CA. After that I turn to conceptualizations of practice and practicality in ethnomethodology (Section III) and CA (Section IV). The two sections will give a sense of the concept of practice both in its value for social theory and as an essential object of study.

## Some uses of “practice” and “practical” in ethnomethodology and conversation analysis

In his *Studies in Ethnomethodology*, Harold Garfinkel uses the term “practice” and “practical” frequently. In his preface, he says:

Ethnomethodological studies analyze everyday activities as members’ methods for making those same activities visibly-rational-and-reportable-for-all-practical-purposes, i.e., “accountable,” as organizations of commonplace everyday activities. The reflexivity of that phenomenon is a singular feature of practical actions, of practical circumstances, of common sense knowledge of social structures, and of practical sociological reasoning (…). Their study is directed to the tasks of (…) discovering the formal properties of commonplace, practical common sense actions, “from within” actual settings, as ongoing accomplishments of those settings (Garfinkel, 1967, pp. vii-viii).

In the quote, Garfinkel speaks of the relevance of the intelligibility of activities for practical purposes. He also mentions the practical modality of actions, circumstances and (sociological) thinking and states formal characteristics of practical actions of common sense. Garfinkel speaks not only of the practical modality of different kinds of social phenomena, but also of practices. In his view, however, these are never stable, but are always in a process of ongoing accomplishment:

Practices consist of an endless, ongoing, contingent accomplishment (Garfinkel, 1967, pp. 1).

In his definition of “ethnomethodology,” he uses both terms and speaks of both “practices” and the “practical modality” of actions. He explains:

I use the term “ethnomethodology” to refer to the investigation of the rational properties of indexical expressions and other practical actions as contingent ongoing accomplishments of organized artful practices of everyday life (Garfinkel, 1967, pp. 11).

Together with Harvey Sacks, Garfinkel reflected on the status of practical modality using the example of practices of natural language use, which they defined at the time as “conversation” (Garfinkel and Sacks, 1970). These reflections were part of the development of the theoretical position of the emerging field of CA. A conversation, they say, like any other social phenomenon, has two “formal properties” (hence the title of their text), as it must fulfill two requirements: (1) it must be realized in an orchestrated way through the use of practical procedures and is necessarily embedded in practical circumstances, and (2) it must be in some way recognizable as a conversation (rather than something else) to those who realize it. To capture this dual character, Garfinkel and Sacks have proposed that, for methodological reasons, any sociological description of a social phenomenon (as realized through practical action) be preceded by the prefix “doing.” This emphasizes not only the practical accomplishment of a phenomenon, but also its recognizability (“accountability”), which is actively achieved by those involved in its practical accomplishment. In the following quotation, they replace the social phenomenon to be described with the symbol “[].”

The expression, [], is prefaced with “doing” in order to emphasize that accountable-conversation-as-a-practical-accomplishment consists only and entirely in and of its work (Garfinkel and Sacks, 1970, pp. 352).

The practical embeddedness of any social activity creates an inescapable “indexicality.” A conversation, for example, is situated in, productive of, and sensitive to, the here-and-now of the conversational situation, and any attempt to remove this indexicality results in a new situation with its own indexicalities. As a co-product of the practical realization of the action, the activity must therefore simultaneously be made recognizable by and for the co-participants as an activity of a certain type. In their text, Garfinkel and Sacks assume a “machinery” that constitutes the practices of “doing” and simultaneously lends each practical doing a moment of intersubjective intelligibility and recognizability without, however, carrying an explicit formulation of it.

What kind of “machinery” makes up the practices of doing [accountably rational conversation]? Are there practices for doing and recognizing [the fact that our activities are accountably rational] without, for example, making a formulation of the setting that the practices are “contexted” in? (Garfinkel and Sacks, 1970, pp. 355).

From this perspective, practices are seen as an intrinsic feature of the procedural accomplishment of social phenomena as meaningful objects. Due to their dual property of being practical and recognizable at the same time, they are not seen as situated in a stably given external context, but rather as themselves creating the setting, or context, that makes them understandable.

CA has taken the concept of “practice” further in its analytic effort to identify the implicit organization of social phenomena: How are they procedurally accomplished and made recognizable in detail by and for co-participants? The empirical program of CA, as advanced by Emanuel Schegloff, proceeded to examine the details of all kinds of conversational practices, thus identifying, step by step, individual pieces of the mosaic that might eventually yield a complete picture of human sociality. This complete picture sheds light on how sociality functions as an implicit organization of the social in general. As Schegloff says, a “web of practices” constitutes the infrastructure of social life, and practices, as distinct entities, are the smallest units of investigation. He asks:

What is this web of practices that serves as the infrastructure of social institutions in the same way that a system of transportation serves as the infrastructure for an economy, that is so transparent that it is opaque, whose omnipresence and centrality make it a-if not the-core root of sociality itself? (Schegloff, 2007, pp. xiii).

Schegloff’s research program of studying practices as the smallest units of sociality is prominent in current CA. But does this almost reifying conceptualization represent a fundamental shift in the understanding of “practice” and “practical” from ethnomethodology to CA? This will be discussed in the further course of this text, which begins with a look at the history of the concept of practice.

## History and variation of conceptualizations of “practice”

In the first theoretical discussions in Antiquity, the noun “practice” was used to emphasize the central ambiguity of human action that, on the one hand, is based on freedom of choice, but, on the other hand, takes place in concrete situations conditioned by external constraints of the real world-social, temporal and material. Aristotle (2004, pp. 3–4, 104–106) contrasts “practice” by two other modes of action: *theory* and *poiesis*. *Theory* deals with the unchangeable and necessary, while *practice* and *poiesis* shape the changeable and contingent. *Poiesis* is a means to an end that leads to an object distinct from the activity, but *practice* is an end in itself. According to Nussbaum (2001, pp. 302–306), Aristotle claims that human beings respond through practical wisdom to practical situations, which is characterized by three features:

1. Mutability: Practice is historically rooted and not supported by nothing more permanent than the ongoing world of human social practice. Since practices change over time, they are capable of surprise. Therefore, the practical actor must always use reason in improvisational and conjectural ways.
2. Indeterminacy: Practice is complex and contextually diverse. Practices must be applicable to a variety of contexts, and this requires the situationality of appropriate choices. The practical actors are obliged to adapt their choices to the complex requirements of specific situations, taking into account all contextual features.
3. Particularity: In every situation, the practical actor has to deal anew with the occurrence of features that are repeatable in themselves in an infinite number of combinations, but which make the complex overall situation a non-repeatable particularity. Particularity emphasizes the unrepeatability of the situation.

In Aristotle’s conception practical wisdom refers to given resources (such as rules) only as rough guides, since the main characteristic of practical wisdom is to be responsive, flexible, ready for surprises, and inventive in improvisation. Central to practical wisdom is the ability to recognize and respond to salient features of a complex situation creatively. This aspect of freedom in praxis is also present in other philosophies such as, prominently, Kant.

Beginning with Hegel, practice was reconceptualized as being carried out in a non-representational, immersionist way. This aspect of an absorbed coping was expanded by Heidegger, who presents the “practical” concern as the original mode of being (Heidegger, 1996, pp. 327). Theory, as *absence* of praxis, is therefore in constant danger of being deficient.^1^ In view of Heidegger (1987, pp. 86; translation modified), “praxis does not mean mere activity and actualization; rather, such activity is grounded in the accomplishment of life itself.” Therefore, practice is crucial for the living being: the accomplishment of existential stability (Heidegger, 1987, pp. 86–87). Embedded in, and related to, the environment that it co-constitutes, practice takes place within, and constantly shapes, a horizon of looking through and looking ahead (Heidegger, 1987, pp. 87). Heidegger combines the anti-representationalist idea that actors are immersed in their doing, involving body and mind, with the importance of temporality, especially anticipation and continuous adaptation to ever-changing material circumstances. For him, the essential function of practice is stabilization.^2^

Marx applied the notion of practice to society in a more encompassing way, which, he says, emerges in long-term historical processes of aggregated individual practice. In this way, “real, corporeal man” creates “an *objective world* by his practical activity” (Marx and Engels, 1988, 153, pp. 76–77).

The different philosophical approaches have thus understood the practical conditionality of human social life in different ways. Aristotle asserts that the instance mediating between the two poles of freedom and constraint is practical wisdom. Since practice is constantly confronted with mutability, indeterminacy, and particularity, it responds on the basis of situation-sensitive practical wisdom that is informed by individual experience and cannot be captured in universal terms. Hegel and Heidegger advocate an anti-representationalist conception of praxis that assumes only a loose coupling between intentional choices and realized actions. They view practical actors as immersed in their practice. In Heidegger’s existential perspective, praxis is seen as the accomplishment of life and as securing its stability. Marx emphasized the historical and social dimensions of practice. For him, humans as species-beings create and recreate a constantly changing objective world through their embodied practical activity. Together with the idea of an only loose coupling of intention and action and the emphasis on temporal and material constraints, the conceptualization of practice as social and permanently reshaping the objective world lays the foundation for later ideas of indeterminate practical self-organization in the sociological theory of the 20th and 21st centuries. Here, the idea of practice filled a theoretical gap insofar as established sociological theories usually assume that either transcendental structures that lie beyond the horizon of the individual (e.g., Blau, 1977) or universally rational considerations and intentions (e.g., Coleman, 1990) determine social action. In contrast, practice theory claims the primordiality of practice over both structure and intention (e.g., Marx, 1970, pp. 122; Schatzki, 2001, pp. 9), thus avoiding to implicitly presuppose the orders whose emergence they want to describe. Practice theory holds that individuals and their intentions as well as structures and institutions are products rather than causes of practice. From this perspective, there is nothing social “beneath,” “above,” or “behind” practices: No structure or system assembles or determines practices. What there is in social life takes place exclusively in practices. The resulting question for this non- or post-metaphysical approach is how practices themselves are stabilized so that they do not arise entirely by chance.

The different versions of sociological practice theory that circulate today explain the sources that provide for the continuity of social order differently: In Bourdieu (1990, pp. 53–55), the habitus as a socialized system of dispositions embodied by human actors feeds into practice and serves as a hinge between the past-history objectified in institutions-and the present-social action. In relation to the latter as being both free and constrained, practical logic is anchored in the dialectic of individual action dispositions and instituted means of action. For Bourdieu (1990, pp. 18), practical logic is embodied in “motor schemes and bodily automatisms” of the habituated body as practical sense (Bourdieu, 1990, pp. 69). The practical sense serves as durably installed generative principle of regulated improvisations and reactivates the sense objectified in institutions. In the model of Bourdieu (1990, pp. 57), the “intentionless invention of regulated improvisation” of practice solves the problem raised by Aristotle that each new situation has unrepeatable particularities.

Giddens (1979) views rules and resources as factors that inform and stabilize practice. When engaging in practices, actors refer, on the one hand, to shared knowledge about rules and conventions. On the other hand, the body and its capabilities serve as resources in which the ways of doing things are stored in the form of “memory traces” (Giddens, 1979, pp. 64). Taken together, they ensure that practices develop into forms of “routine action” that reduce cognitive effort and anxiety, because, as Giddens (1979, pp. 218) says, they are “strongly saturated by the ‘taken for granted’.” An example of this is ethno-methods, which are latently accepted by the parties “however much they involve a labor of reflexive attention” (Giddens, 1979, pp. 218). The solution of Giddens (1979, pp. 18) for the problem of the non-repeatable particularities of ever-new situations is “rule-governed creativity,” which, however, consists in the application of fixed, given rules and “is at the same time the medium whereby those rules are reproduced and hence in principle modified.” The reason of Giddens (1979, pp. 57–73) for the relative persistence of the taken-for-granted is that in his view practice is informed by practical consciousness, i.e., tacit knowledge embodied in what actors “know how to do” that is skillfully applied in the enactment of courses of conduct, but which the actor is not able to formulate discursively. Though Giddens (1979, pp. 25) insists that “the *reflexive monitoring of conduct* (…) is central to human activity,” he sees the “basic significance of practical consciousness in social reproduction” (Giddens, 1979, pp. 256).

Thus, from both Giddens’ and Bourdieu’s perspective, unlike Aristotle’s, it is not stability and continuity but social change that needs to be explained, since both the habitus and practical consciousness tend to reproduce social structure. Both advocate an anti-representationalist approach to practice that emphasizes its habituated, routine-like, and non-intentional character. Both also downplay the role of intentions in favor of routine practices, thus advocating a notion of socio-practical self-organization.

Other, more recent practice theories present similar types of explanation: In Shove’s approach (Shove et al., 2012), persisting sets of materials, meanings, and competences steady the social. For Latour (2005), network-like assemblages of hybrid entities that generate action can be reconstructed in a flat ontology that does not discriminate between reflexive and non-reflexive participants. And in Schatzki (2002), practices are organized by common understandings, teleo-affectivities (ends, tasks, and emotions), and rules. Thus, these new approaches also assume a tacit reproduction of the existing through practices informed by rules and bodies of knowledge, and neglect the relevance of practical wisdom that responds competently and knowledgeably to the inevitable mutability, indeterminacy, and particularity of the new.

These newer conceptions also differ in their estimation about the “size” of practices, whether they refer to larger historical processes, as in Marx, or to small units constituting actions, as in CA. For Schatzki (2002, pp. 245), practices are larger “bundles of doings and sayings,” while Nicolini (2013, pp. 2) considers them as “vast arrays or assemblages of performances (…) knotted together in such a way that the result of one performance becomes the resource for another.” Society here appears as a kind of perpetuum mobile.

Although practice theories aim to demonstrate the primacy of practice over structure and intention, the approaches discussed here conceptualize rules and embodied knowledge as structural givens that determine practice because they are seen as shared by actors in identical way from the outset. In particular, the questions of where the commonality and identity as well as the recognizability of these different resources and guidelines come from, how they are permanently reproduced, and how they are implemented homogeneously remain open. These are questions that ethnomethodology dealt with from the very beginning.

## Concepts of “practice” in ethnomethodology and conversation analysis

As has been shown, Aristotle poses the problem of social order in a radical way. For him, social situations are always new, so that social actors are always confronted with the question of how to act under the given circumstances. From his perspective, experienced actors act with the help of practical wisdom that allows them to deal competently with the mutability, indeterminacy, and particularity of ever-new situations. Practice here means the self-organization and openness to future action. The scholars following this discussion emphasize that practice is an anti-representational mode of activity, and in which the actors are immersed in their actions (e.g., Hegel and Heidegger). In sociology, theories of practice that emphasize the routine character of action (e.g., Giddens) follow on from this: for them, the problem of the permanent novelty of social situations does not arise; rather, social situations are understood as recurring and repetitive. Other approaches (e.g., Marx and Bourdieu) emphasize the historical growth and tradition of social forms of activity and see their stability and continuity in this. These approaches also emphasize the sharedness and thus recognizability of practical forms as resources for intersubjectivity, social coordination and order. Aristotle’s original problem was thus increasingly solved by assumptions about given structural conditions that guide and inform practice. However, ethnomethodology and CA take different position.

### Ethnomethodology: the practical character of sociality

Garfinkel’s notions of “practice” and the “practical” were influenced by different authors including Wittgenstein, Heidegger, Merleau-Ponty, Gurwitsch, and Schütz. A surprising early reference of Garfinkel (1956) is to the Polish philosopher Tadeusz Kotarbinski,^3^ who was influenced not only by Marx, but also by Ludwig von Mises’ economic praxeology. Garfinkel had even considered using the term “neo-praxeology” as alternative for “ethnomethodology” (Garfinkel in Hill and Crittenden, 1968, pp. 10). He recommended a text on Kotarbinski, from which the following quote is taken.

The main task of [Kotarbinski’s] praxeology is the search for similarities of successful methods in many different domains of action. For example, the method of delaying an attack is not specific for military strategy or for games. We apply it with success in oral disputes, and in art when a composer puts his most striking effect at the end of his composition as did Beethoven in the Ninth Symphony. To say for example that a scientist improves on his chance of a success by keeping in mind the principle of changing the plan of his work during the course of the work in view of results already obtained is to say a truth. Praxeology does not attempt to teach anything new about these materials. It rather records the methods applied by workers (…). It merely records, systematizes, and analyzes the existing techniques. The practical gain from praxeology is (…) in making explicit the methods already in use. Practical values are different from ethical values (…). There are cases when more than one person is the agent, as when two persons play a four-hand piano piece, or when one person prepares the material to the further work of another person. There are also cases when no single one of the collaborating persons can rightly be considered a perpetrator of the product, e.g., when several persons are pushing a car (Hiz, 1954, pp. 239).

In Kotarbinski’s conceptualization, praxeology records and makes explicit successful practical methods applied by practical experts in various fields of action. These practical methods can be performed by individual actors or by more than one person, and even if no individual person can be considered the actor. One practical method Kotarbinski mentions is “delaying.” It is used in many different fields, one of which is well-known to scholars in CA: preference organization, where delay occurs when, for example, invitees decline an invitation. Kotarbinski was also interested in popular practical knowledge, as expressed in proverbs and rules of thumb, among which Garfinkel includes practices of “ad hocing” such as “*et cetera*,” “let it pass,” “unless,” or “factum valet” (Garfinkel, 1967, pp. 20–21).

Other important theoretical dimensions of Garfinkel’s concept of practice are known:

1. Natural Attitude: In our “attitude of daily life,” as Garfinkel says, echoing the “natural attitude” of Husserl and Schütz, we are interested in getting things done and not in the ontologies of those things or the epistemology of our knowledge about them. The “natural attitude” of everyday life, according to Schütz, is a pragmatic stance oriented toward practical purposes and relevancies, suspending doubt. This includes the assumption that the world is from the outset an intersubjective world, “common to all of us” (Schütz, 1945, pp. 534). In ethnomethodological texts, the term “practical” is used in this sense when speaking of conditions that constrain the realization of actions in everyday situations. Garfinkel (1967, pp. 7) speaks of actors’ reflection on these conditions in terms of non-theoretical principles such as “for practical purposes,” “in light of this situation,” or “given the nature of actual circumstances” that guide practical action. In one of the rare instances in which Garfinkel cites his sources, he refers to Schütz in regard to his notion of practice and the practical (Garfinkel and Sacks, 1970, 341–342).
2. Temporality: The “aphorism” of ethnomethodology is to not treat social phenomena as objective facts, as “things,” like Durkheim, but as practical productions, as achievements, as ongoing accomplishments of the members. Garfinkel (1967, pp. 182) is interested in the details of “the steps whereby the society hides from its members its activities of organization and thus leads them to see its features as determinate and independent objects.” Social reality does not exist independently of the practical activities from which it emerges. “Practice” refers to the procedural accomplishment and achievement of situation-specific social particularities of interaction. Each realization of an action represents a selection from other possibilities of action. While social actors are engaged in practice, time moves on incessantly and relentlessly; and they cannot step out of this clockwork: there is “no time out,” “no possibility of evasion,” and “no hiding” (Garfinkel, 2002, pp. 118). The time a member has to weigh up different alternatives is limited and usually extremely short (Garfinkel, 2002, pp. 118). The actors and their situational perspective experience a constant pressure to make choices in regard to further actions. Garfinkel (1967, pp. 12) calls this problem “the practical question *par excellence*: ‘What to do next?’” Although no references to Aristotle are made, his treatment of practice resonates in Garfinkel’s words, especially the ideas of openness to the future (indeterminacy) and constant change (mutability). Garfinkel (1967, pp. 11–18) is also interested in understanding how actors make choices in the here-and-now of their practical situation.
3. Within-ness and indexicality: Garfinkel was dissatisfied with contemporary approaches to rationality (e.g., from the emerging rational choice theories) that defined criteria of rational action as absolute and universal and not, as Garfinkel intended, from *within* the situation and the perspective of the actors. Since actors are inevitably part of their situation and act from within it, Garfinkel called them “members.” Members of a society have an interpretative competence that enables them to practically reason about the particularities of their situation as a practical basis for their decisions. People in everyday life act as “practical methodologists” (Garfinkel, 1967, pp. 180) who solve decision-making issues with the help of everyday knowledge and “practical reasoning” (Garfinkel, 1967, pp. 11–31). This idea resonates with Aristotle’s concept of practical wisdom. Because they are situated in a specific here-and-now, practical activities are *indexical*: They refer to, and thus constitute as relevant, contextual elements of the particular situation (think of Aristotle’s notion of particularity). Indexical elements have a practical *in situ* meaning and rationality.
4. Accountability: Since nothing external determines the practical situation of here-and-now such as causal forces or an external context, Garfinkel argues that external variables are only relevant for the situation when members “discover” them in their situation and make them relevant and accountable. When members do something, they, identically with, and simultaneously to, their doing, produce, evoke and thus accomplish the contexts and “practical circumstances” (Garfinkel, 1967, pp. 172–185) that make their doing understandable. Thereby, “phenomena of order [that appear external and objective] are identical with procedures for their endogenous production and accountability,” he says (2002, 72). This implies that natural language is intrinsically embedded in practical action; the latter is never an outer addition to an otherwise silent, or tacit phenomenon. Instead, when we accomplish recursive activities such as repairs, glossings, or formulations, they occur *within* the temporal process and *within* the situational indexicalities and evoked contexts. In practice, things explain themselves practically.

As we see, Garfinkel’s concept of practice differs from the sociological theories of practice mentioned above in several respects. On the one hand, Garfinkel rejects the idea of an anti-representationalist immersion in practice, but also does not commit himself to intentionalist notions of always rational choice, as advocated in rational choice programs or in Schütz’s egology. Accordingly, neither theories of routine (Giddens) nor of action projection, controlled by the ego (Schütz), are capable of explanation for him. Rather, Garfinkel maintains the Aristotelian thought of praxis as field of freedom that is conditioned by social, material, and temporal constraints, but which cannot be explained by universally valid rules. According to Garfinkel, universal rules cannot explain practical action, as they cannot anticipate and regulate their application under all conceivable conditions. They therefore present the actors with the constant problem of interpreting the concrete situation of rule application in terms of which possible rules apply to them and how these are to be applied. The application of the rule itself cannot be part of the set of rules, as this would lead to an infinite regress. Rule following is therefore always provisional and constantly adaptable and changeable. In most cases, rules are only “discovered” after action has been taken, when they need to be explained and justified, for example in the event of disruptions. In his interpretation of rule following, Garfinkel is close to Wittgenstein. It also becomes clear why he had sympathies for Kotarbinski.

By practice, Garfinkel particularly addresses the ongoing accomplishment, indexical here-and-nowness, and situational contingency of human action, as did Aristotle. He also doubts that the assumption of other shared resources (especially knowledge) can explain the social character and intersubjective validity of practice. Rather, his attempt is to also theorize the sharedness of resources praxeologically, i.e., as an effect not a cause of practices. Like Marx and Bourdieu, Garfinkel abandons entirely the idea that practices principally originate in individuals. But if practice is fundamental even to the sharedness of resources, and social actors as individuals or groups or as members of collectives or cultures are also theorized as produced by practices (*cf.* Lynch, 2012), then the question arises what it is that stabilizes and continues the social.

The problem of connecting the primordiality of practice with the *a priori* of intersubjectivity can be understood by referring to the gestalt phenomenology of Aron Gurwitsch, which had an enormous influence on Garfinkel (2002, 2007, 2021). Especially in his texts published in the 2000s, Garfinkel repeatedly used cryptic phrases about the practical accomplishment of social activities. Social activities, he says, are “composed endogenously, in-and-as-of-their-lived-temporal-in-course sequentiality” and achieved in “‘strings’ of coherent contextural constituents of lived orderlinesses in practices of ordinary society” (Garfinkel, 2007, pp. 42). Expressions like these become understandable only in the light of Gurwitsch’s Gestalt phenomenology. For Gurwitsch, people are absorbed in constant configurations and reconfigurations of situations that they at the same time perceive and co-produce. In this perspective, practice originates in situations that provide affordances and opportunities for members to participate while these members themselves produce these situations. This is the essential departure from an individualistic perspective of practice, which nevertheless recognizes the mutability, indeterminacy and particularity of practice in the sense of “self-organization” (Garfinkel, 1967, pp. 33).

**Figure 1:**
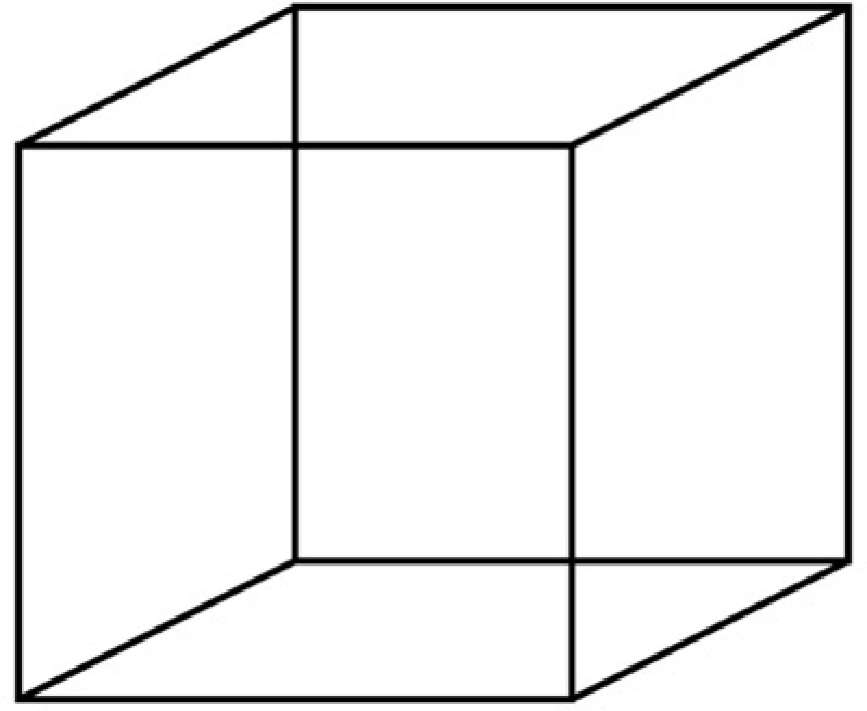
Necker cube.

The background to this conception is that Gurwitsch, in contrast to Schütz, advocates a “non-egological conception of consciousness.” He claims that the recognition of perceptual objects is not actively controlled and rationally penetrated by the ego, but is stimulated by the phenomenon as it appears to consciousness. Gurwitsch illustrates this idea with reversible figures such as the Necker cube (Figure 1).

Gurwitsch notes that this figure defies our voluntary focus of attention. When we actively try to see, for example, the bottom left corner of the cube as the back or front, we are often disappointed because we cannot fully control our perception. Only rarely do these figures appear as an active achievement of our voluntary mental perceptual action. Much more frequently, they change their configuration without our intention and will. The coherence of the perceptual elements as parts of a coherent whole is not actively organized by us as perceivers. This is why Gurwitsch calls their organization *autochthonous*: Perception is self-organizing. Gurwitsch emphasizes the autonomy and self-regulation of meaning structures and meaning processes as they appear to consciousness in general (*cf.* Meyer, 2022).

Rather than using Gurwitsch’s term “autochthonous” to refer to the property of perceptual qualities being independent of the perceiving ego and their relevancies, Garfinkel uses the term “endogenous” (Garfinkel, 2002, pp. 176). In contrast to the anti-representationalist immersionism of some of the theorists presented above, Gurwitsch and Garfinkel emphasize conscious action and perception, which they consider to bei neither routine nor completely under the control of the ego.

**Figure 2:**
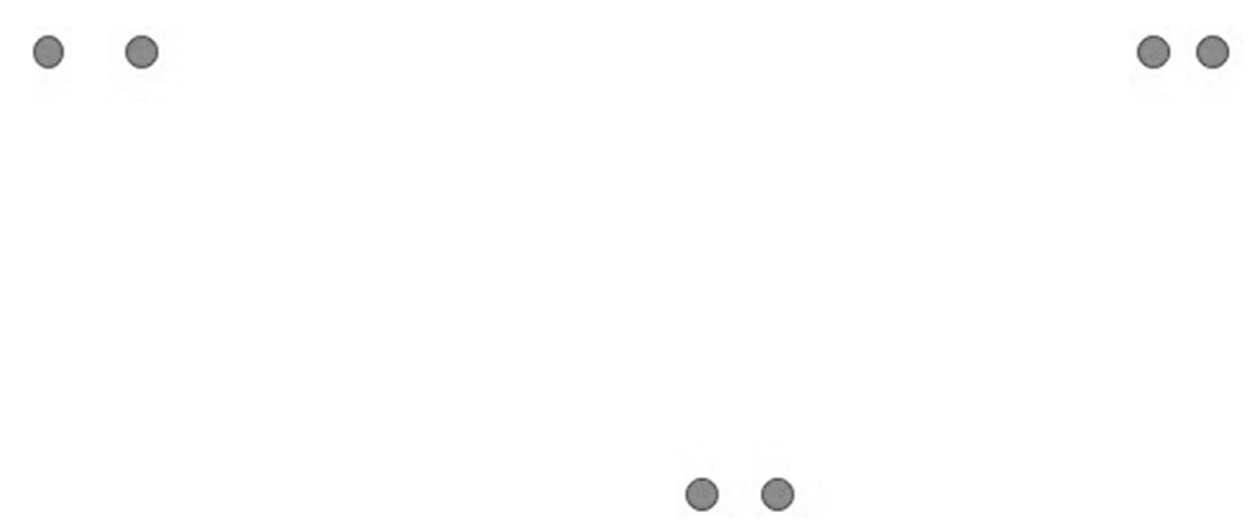
Three pairs of dots.

In Gurwitsch’s theory, gestalt contexture encompasses three parts, metaphorized by a circle, two of which are interesting for us here: The theme occupies the center of this circle, it stands in the thematic-field, which forms the area of the circle. The *theme* is organized by “a group of data” (Gurwitsch, 2010b, pp. 29), creating an internal *gestalt coherence*, in which each component is related to all other components and has a “functional significance” for the whole. Below is a typical figure that Gurwitsch uses to illustrate his ideas on gestalt perception: A pair of dots that are in a reciprocal relationship of left or right, above or below, far or near (Figure 2).

**Figure 3:**
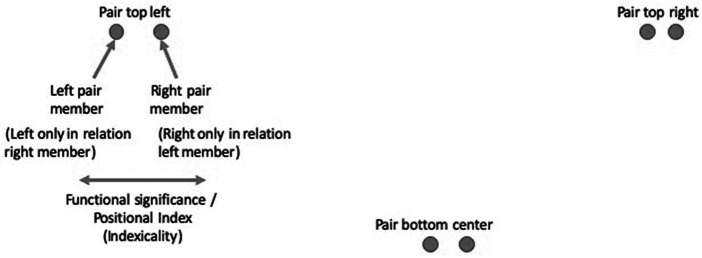
Three pairs of dots explained.

We see three pairs of dots arranged at different distances from one another. The pair at the top right is the closest, the pair at the top left is the furthest apart. Gurwitsch says, “the indexical terms ‘neighborhood,’ ‘relative proximity,’ ‘moderate proximity,’ ‘immediate surroundings,’ ‘wider surroundings,’ ‘close by,’ ‘next to,’ and others designate phenomenological qualities and not distances in a merely quantitative sense” (Gurwitsch, 2010a, pp. 218–219) (Figure 3).

Each of the pairs has a left and a right member. However, the left member is only to the left within the constellation of the pair itself, not in absolute terms. Similarly, the right member is only to the right of the left pair member of the pair. If we were to add a dot to one of these pairs, e.g., to the left, the dot that is currently to the left would become a middle dot of a triple, and the whole gestalt would re-configure itself. It is no coincidence that Garfinkel uses the term member in this double sense-even members of society can be seen as members of ever-changing gestalt contextures that only have meaning as wholes and whose members only have meaning relative to the gestalt whole and to other members.

According to Gurwitsch, it is therefore an indexing structure, in which the individual dots do not possess an intrinsic, but a context-dependent, functional significance. They produce a positional index that only applies to the internal gestalt structure of the pair. It has an indexical structure “from within.” It is the internal *constellation*, the gestalt contexture that creates meaning, not the aggregation of individual elements that are meaningful in themselves. Philosophically, both Gurwitsch and Garfinkel (together with Wittgenstein and others) advocate a radical semantic holism. Each dot simultaneously “incarnates” and “reflects” its role within the gestalt (here: the pair) (*cf.* Garfinkel, 1967, pp. 1). The adoption of semantic holism involves the “abandonment of the constancy hypothesis” (Garfinkel, 2021, pp. 20), which assumes stable functional significances and meanings of elements of gestalt contextures as well as variables of social situations (e.g., members, procedures, resources, and rules), conceptualizing them instead as entirely contextually determined.

We have said that the individual dots do not have an intrinsic meaning, but a context-dependent, functional meaning. However, the context on which the functional meaning of each individual dot depends-the pair-is not external to the individual dots, but is generated by these dots themselves (Gurwitsch, 2010b, pp. 331). Details, totality, and context thus constitute one another. Since details are context constituting, context cannot be viewed as external container. The context that only acquires unity through its *relevancy* for the theme is called *thematic field* by Gurwitsch.

According to Gurwitsch, each element has a “positional index” that establishes a particular thematic field as its context that makes it understandable. Garfinkel (2021, pp. 25) re-terms “functional significance” as “organizational” or “figurative details.” These details, according to Garfinkel (1967, pp. 40), mutually point to, and elaborate, one another, creating the “essential indexicality” (e.g., Garfinkel, 2007, pp. 43ff) of such phenomena.

However, Gurwitsch did not only develop his theory of “gestalt contexture” using the example of visual forms, but also situated it in time. His example is music, in which we can observe a dynamic, ever-changing gestalt contexture that reconstitutes in our experience each moment anew. As with the examples of dots, its individual sense-data do not have stable core meanings, but interact with their immediate, self-generated context. This context is sequential, and tones are parts of melodies that form a whole. When the context changes, the meaning of the individual sense-data also changes. This means that we can only participate in a meaningful practice *from within*.

Although Gurwitsch’s model is not itself practice-theoretical, it emphasizes the temporal process of ongoing interaction between perception and perceived objects. It shows how phenomena self-organize sequentially in time by indexing and appresenting absent or imminent elements that complete the perception. Objects of perception are therefore meaningful from within, because they constantly self-constitute and self-supplement based on past experiences.

Garfinkel transferred this line of thought to the social sphere, which cannot be viewed from the outside like Gurwitsch’s gestalt contextures. Rather, both sociologists and laypeople, are members of these gestalt contextures in a double sense: they are active participants and constitutive parts. Garfinkel and Livingston (2003, pp. 26) agree that in social life “contexturally coherent Things are massively prevalent, recurrent, each in coherent witnessed details that are seen but unnoticed.” In the realm of the social, contexturally coherent things are far more complex than in the gestalt experiments on which Gurwitsch relied. For in the case of social phenomena, the “produced coherence of organizational objects” (Garfinkel, 2021, pp. 30) is *interactionally* and *practically* “achieved.” The objects of sociology are constituted through constantly changing “actions and practices” (Garfinkel, 2021, pp. 21). The most important feature of social objects is therefore that they are not only perceived, but also, and often simultaneously, produced. Moreover, they are produced in interaction to be witnessable, observable-reportable, practically and in an embodied, “incarnate” and “reflexive” manner (Garfinkel, 1967, pp.1), which Garfinkel famously called “accountable.” For the “gestalt contextures” in the realm of the social, this implies that they are necessarily dynamic, temporal, and unfolding, and therefore always transient and mutable, they can never be returned to Garfinkel (2021, pp. 26–27).

As initially quoted, Garfinkel (1967, pp. vii-viii) argues that social activities are reflexively accomplished within a field consisting of practical actions, practical circumstances, common sense knowledge of social structures, and practical sociological reasoning. Since members are constantly engaged in practical reasoning, the gestalt contextures they perceive are organized not only in temporal sequences but also in hermeneutic cycles or documentary manner. This is the case when, as Garfinkel (1967, pp. 78) puts it,

“an actual appearance” is treated “as ‘the document of’, as ‘pointing to’, as ‘standing on behalf of’ a presupposed underlying pattern. Not only is the underlying pattern derived from its individual documentary evidences, but also the individual documentary evidences, in their tum, are interpreted on the basis of ‘what is known’ about the underlying pattern. Each is used to elaborate the other.”

Or, to put in Gurwitsch’s words: There is a constant switching between themes and thematic fields, since themes indexically refer to thematic fields as their contexts, and thematic fields refer to themes as their typical details. Since gestalt contextures operate in time, they appresent possible, expectable nexts. Each of these indexical references are essentially *haecceitic*, i.e., unique and specific, says Garfinkel (2002). For the concept of practice, this implies that through a systematic perspective on social phenomena “from within” these haecceitic indexicalities must be recognized as particularities (see Meyer, 2022, pp. 133–138).

Garfinkel has shown in various case studies of staff at a Suicide Prevention Center, staff using files from psychiatric hospitals, graduate student coding psychiatric records, jurors in court, a transgender person managing gender reassignment surgery, and professional sociological researchers, that they are all constantly engaged in “practical sociological reasoning” as members. Practical sociological reasoning means that when dealing with everyday sociological matters (such as determining guilt or suicide), choices and selections are made by relying on commonsense knowledge of social structure as thematic field, while the members’ “concerns are for what is decidable ‘for practical purposes,’ ‘in light of this situation,’ ‘given the nature of actual circumstances’” (Garfinkel, 1967, pp. 7), thus specifying the details of the theme. This is also where Kotarbinski’s practical knowledge, as expressed in proverbs and rules of thumb, comes into play. Practical reasoning therefore involves reasoning about the general properties of indexical expressions and other practical actions and their respective uses in the here-and-now (1967: 4, 11). It can be understood as Aristotle’s practical wisdom in action.

Together with “practical action” and “practical circumstances,” “practical reasoning” is part of a “triangle” of practical activities with which Garfinkel answers the question of how social order is practically accomplished and how stability and rationality are maintained, although their accomplishment remains undetermined by external variables.

Practical circumstances refer to “organizationally important and serious matters” such as constraints, resources, goals, excuses, opportunities, or tasks. They relate to the “texture of relevances” (Garfinkel, 1967, pp. 174), the thematic field as it exists in the situation. As Garfinkel has shown, the transgender person Agnes has learned to manipulate the practical circumstances so that she as part of them appears as naturally female, thus exploiting the “préjugé du monde” that circumstances are external, objective structures. This is because, as Garfinkel (1967, pp. 8) says using the example of the staff of the Suicide Prevention Center he studied, members are interested in assuring “the unequivocal recognition of ‘what really happened’.” They are “‘not interested’ in studying practical actions and practical sociological reasoning” as a topic. Rather, “members take for granted that a member must at the outset ‘know’ the settings in which he is to operate” if his (or her) practices are to address the particular features of these settings. They do not take into account the fact that their practices are constituent features of the circumstances they are part of.

As a result, their findings are only seemingly discovered, while they have in fact practically constituted their discoverability in the first place (1967, pp. 9). This is why Garfinkel (1967, pp. 115) concludes in his study on jurors “persons, *in the course of a career of actions,* discover the nature of the situations in which they are acting, and (.) the actor’s own actions are first order determinants of the sense that situations have, in which, literally speaking, actors *find* themselves” (orig. emph.). Practical reasoning can therefore be understood as a procedure of discovery “from within” the indexical situation. Accordingly, any presupposed consensus (belief, norm, value, and rule) of a particular moment can be “retrospectively reread to find out in light of present practical circumstances” what it “‘really’ consisted of ‘in the first place’ and ‘all along’” (Garfinkel, 1967, pp. 74).

As for their function for society, Garfinkel (1967, pp. 182) considers practices as methods “whereby the society hides from its members its activities of organization and thus leads them to see its features as determinate and independent objects.” In other words, practices usually remain implicit, because they thereby maintain the “préjugé du monde” of their members that society and its instances (conversations, institutions, and institutional orders) are external, objective and “immortal” (Garfinkel, 2002). This paradoxical situation, in which actors actually practically accomplish the phenomena of order that they, in their natural attitude, experience as external and objective, is the reason why Garfinkel speaks of the “discovery” of these phenomena of order:

“Persons, *in the course of a career of actions,* discover the nature of the situations in which they are acting, and (.) the actor’s own actions are first order determinants of the sense that situations have, in which, literally speaking, actors *find* themselves” (Garfinkel, 1967, pp. 115, orig. emph.).

It is thus in the nature of practices that they discover what they themselves produce as orienting circumstances.

Gurwitsch has posited the same concept of discoverability against anti-representationalist and routine-related conceptions of practice in relation to material objects. He says that “practical reckoning” and our “specific practical experience” must be distinguished from routine and habituation, because new situations can only be mastered on their basis (Gurwitsch, 1977, pp. 65). In practical action, he says (Gurwitsch, 1977, pp. 79), I constantly look at and find manifold references in my environment. “I thus ‘discover’ them while placing myself at their disposal and following them.” Thus, “when I gear into the situation and comport myself according to the ways prescribed by it, the indexical contexture and situation become visible” to me (Gurwitsch, 1977, pp. 79). Through my actions, the surrounding world, as indexical contexture, is indicated as discoverable (Gurwitsch, 1977, pp. 73). Therefore, according to Gurwitsch, all “‘seeing’, ‘perceiving’, ‘noticing’, ‘knowing’” are “in service of ‘being in the situation’” and “are themselves but moments of it” (Gurwitsch, 1977, pp. 85).

In contrast to practice theories that base their argumentation on strong notions of routine and non-representationalist immersion in the situation, Garfinkel emphasizes the importance of the aspect of freedom by including concepts of situation-sensitivity as well as creativity (“artfulness”) with this triangle of “practical action,” “practical reasoning,” and “practical circumstances.” This comes close to Aristotle’s notion of practical wisdom. For Garfinkel does not abandon the idea of the reflecting actor in favor of practical immersion and absorption, but connects rational action to haecceitic situational affordances and reasons. To put it with Heritage (1988, pp. 128): “Garfinkel concluded that shared methods of reasoning generate continuously updated implicit understandings of what is happening in social contexts-a ‘running index’, as it were, of what is happening in a social event.” However, Garfinkel never saw the sharedness of resources as solution to the question of the “how” of practices and social order. In his view, the sharedness of the methods is rather a result of the practical accomplishment of the social, and a constant problem to be tried out, examined, reflected upon and discovered by members who only emerge relative to other members and to the whole.

### Ambiguous meanings of “practice” in conversation analysis

In ethnomethodology, the term “practice” serves to conceptualize the social in a non-deterministic way: as an ongoing accomplishment of social objects, which is characterized by situational contingency and an indexical here-and-nowness. In this process, variables or semantic details are seen not as stably shared, but as fluid and constantly reorganizing over time. They include all relevant factors present in the social situation: Members, procedures, resources, and rules.

Conversation analysis relies heavily on this orientation and is interested in how social phenomena-such as “a conversation”-despite their mutability, indeterminacy and particularity, can be organized *in situ* by the participants and at the same time made recognizable to them, and this in the course of the action itself, in which this organization rarely becomes thematic, but remains implicit. The aim is to explain this without recourse to variables such as structural determination, routine or rational decision. However, as I will argue, by adopting theoretical concepts that presuppose stable variables (specifically a context-free apparatus that encompasses rules and resources) CA also departs from ethnomethodology and develops an inconsistent concept of practice.

For example, Schegloff says on the one hand, that “sequence organization” is a “practice, rather than [a] fixed structure” (Schegloff, 2007, pp. 201) and that sequences themselves must be seen as practices (Schegloff, 2007, pp. 231–250). From this perspective, conversations do not consist in the expression of intentions or desires, but in “sequential practices and structurings of an interactional project” (Schegloff, 2007, pp. 63). In this usage, practice appears in an ethnomethodological sense as organizational principle and essential character of human interaction and social life in general.

On the other hand, however, Schegloff (2007, pp. 71) says in the same text-to quote some of his formulations-that sequences, such as adjacency pairs, are “built,” “implemented” (Schegloff, 2007, pp. 81) or “produced” (Schegloff, 2007, pp. 162) by “diverse practices” (Schegloff, 2007, pp. 161) that stem from a “range of practices on which (…) speakers may draw” (Schegloff, 2007, pp. 164). His idea is that there is an “underlying range of orderly structures and a set of practices for suiting those structures to the particulars of the moment in which the participants are acting” (Schegloff, 2007, pp. 220), and that furthermore a “range of practices and resources [is] brought to bear in [interactional] trajectories,” occasioned by specific interactional developments (Schegloff, 2007, pp. 193). Practices use and rely on “structural and normative resources” (Schegloff, 2007, pp. 203), of which sequences are one. Thus, for Schegloff (2007, pp. 220), “sequence structure (…) has considerable scope and robustness.” It “should be understood as an organizational resource-a kind of convergently oriented-to set of possible routes-which the participants draw on in charting and incrementally building a joint course of action” (Schegloff, 2007, pp. 220). This is achieved through the use of individually definable practices. Practices are understood here as distinctive means of production, implementation tools or building blocks that are used for the specific design of otherwise generally robust sequential structures. In this perspective, practices seem to have a relatively stable identity as individual entities, and sequences a relatively stable (“robust”) structure.

Thus, while in some places Schegloff (2007) understands sequences themselves as practice or ongoing practical accomplishment, in other places practices are seen as individually selected and clearly definable entities that construct and at the same time draw on sequences as relatively robust underlying structures, which appear external to them. It is this second conceptual orientation that visibly deviates from the previously identified ethnomethodological view of practices. In my view, this inconsistent meaning of the term “practice“, which-as I will show below-is widespread in CA, can be interpreted partly as a consequence of theoretical decisions made in the most cited text of CA authored by Sacks et al. (1974) (henceforth: SSJ). In this text, the authors argue that conversation can best be explained by assuming a “formal apparatus” that consists of “context-free resources,” a “context-free structure” or a “context-free organization” and their “context-sensitive” application (1974, pp. 699, 699 n. 8). With this model, the authors hope to explain why “conversation can accommodate” such a “wide range of situations” as it does empirically, why “it can be sensitive to the various combinations” and why it is even “capable of dealing with a change of situation within a situation” (SSJ, pp. 699). As their wording indicates, the authors were inspired by, but also reframed, Chomsky’s (1965, pp. 17, 63 et seq) notion of “transformational apparatus” as well as “context-free” and “context-sensitive” grammars: Unlike Chomsky they do not see “context-free” and “context-sensitive” as mutually exclusive, but as related, and their formal apparatus is not cognitive but social-procedural. The idea of a formal apparatus, at least for Sacks,^4^ also solves what Chomsky (1986, 51–204) has called “Plato’s problem”: Why is it that

“members of the culture, encountering from their infancy a very small portion of it, and a random portion in a way (the parents they happen to have, the experiences they happen to have, the vocabulary that happens to be thrown at them in whatever utterances they happen to encounter), would come out in many ways much like everybody else and able to deal with just about anyone else. (…) Tap into whomsoever, wheresoever, and we get much the same things” (Sacks, 1984, pp. 22).

Sacks’ answer is that “culture is an apparatus for generating recognizable actions; [and] the same procedures are used for generating as for detecting” (Sacks, 1995, pp. 226).

Sacks here follows Garfinkel’s famous “identity theorem”: Practices that constitute meaning and practices that interpret meaning are identical (Garfinkel, 2002, pp. 72). However, as we have seen above, the idea that the formal apparatus necessarily needs to encompass a context-free core, is not supported by Garfinkel (1967, pp. 40), who thinks that contexts and their details mutually elaborate one another and that practices, therefore, are “context-producing.” Just as there are no practice-free contexts (since contexts are produced by practices), there are also no context-free practices (since the meaning of practices is produced by contexts that are produced by practices).

SSJ focus on turn-taking as example of the implicit constitution of social order in the course of the social activity itself, which is accomplished through the sequential concatenation of practices that implicitly shape social life. Although there are no predetermined structure and no explicit rules of everyday conversations with regard to the choice of topic and the change of speaker, and no one knows in advance what each of the interlocutors will say, how long they will speak or who will speak next, the participants nevertheless create a comprehensible development of topics and an orderly and recognizable sequencing of the conversation through, in the course of, and identical with, their actions. In order for this to be achievable, SSJ claim that “major aspects” of its organization, “are insensitive to such parameters of context [as places, times, and identities], and are, in that sense, ‘context-free’” (SSJ, pp. 699 n. 8). However, they also point out that the apparatus must be “sensitive to” the local circumstances and “exhibit its sensitivity” to them (SSJ, pp. 699). The context-free resources are “employed” or “disposed in ways fitted to particulars of context.” But the context-free structure defines “how and where context-sensitivity can be displayed” and “the particularities of context are exhibited in systematically organized ways and places” that are also “shaped by the context-free organization” (SSJ, 699 n. 8). This position is close to the second meaning of “practice” in Schegloff (2007), as analyzed above. Context here appears precisely as an external container, independent from the particular practices that are assumed to occur *within* it.

Not long before the publication of SSJ’s argument, Garfinkel and Sacks (1970, pp. 355) had developed a different argument that is more akin to the first meaning of “practice” present in Schegloff (2007), as analyzed above. Here, an “action” is an “accomplishment” or “work” that is achieved as assemblage of practices (Garfinkel and Sacks, 1970, pp. 342). In their text, Garfinkel and Sacks also discuss the distinction between context-sensitive and context-free expressions in conversation. They argue that in science there is often an attempt to replace context-dependent, “indexical” expressions, which can only be understood from the immediate circumstances, with objective expressions whose meaning is supposedly context-free. However, this leads to an endless regress, because all expressions depend on an order that binds them to the situation of their use (Garfinkel and Sacks, 1970, pp. 360–361). Often, however, social actors are themselves engaged in decoupling expressions from the immediate circumstances and generalizing them, e.g., when they produce “formulations” or “glossings.” Instead of assuming a hidden apparatus that is only accessible to researchers, they propose to use these actor concepts empirically to determine what members themselves (situation-specifically) consider context-free. Their approach thus represents a significant difference to the SSJ model of 4 years later.

The fact that “practice” is used simultaneously for the context-free resources as entities and for the idea of the ongoing accomplishment, particularity and contingency of all resources has led to an undecided position of CA in regard to this term as visible in Schegloff (2007).

The first explicit use of the concept of “a practice” as an entity probably comes from Schegloff (1972). Schegloff’s (1972, 115) outline still echoes the ethnomethodological approach, when he proposes a talk-intrinsic sense of context saying that the “*participants* analyze context and use the product of their analysis in producing their interaction.” However, he also reifies the notion of practice, saying that the production of “a world of specific scenes,” i.e., social reality, is achieved and exhibited through “a set of general formal practices” (Schegloff, 1972, pp. 117). Practices “accomplish and exhibit the particularities of an interaction (…) through general, formal structures” (Schegloff, 1972, pp. 115). These “general, formal structures” form the context-free core of practices and are used to represent the context of the interaction as understood by the co-participants (Schegloff, 1972, pp. 115). From this perspective, he asks programmatically for CA in general (Schegloff, 1972, pp. 115–116): What are the practices that allow conversation “to operate within very tight constraints” *in situ*, while themselves being “the outcome of a general practice and part of a general structure”? What conversational practices are “subject to similar usage,” what are their “kinds of organization,” and how are they “fitted to one another”?

On the one hand, CA insists that interaction partners accomplish social reality practically by continuously observing each other’s actions and utterances in terms of “why that now?” (Schegloff, 2007, pp. 245) and thus adheres to the principle of practical reasoning. On the other hand, CA is particularly interested in the *general* resources with which the actors constitute their actions. In this relation, Schegloff’s “set of general formal practices” is available to the actors as context-free resources and thus as stable units. Theoretically, the assumption of an intrinsic meaning of stable core units is in line with the position of semantic atomism as advanced by Katz and Fodor (1963) following Chomsky in the 1960s. Garfinkel in contrast, advocates a radical semantic holism following Gurwitsch and Wittgenstein that claims the essential indexicality, situatedness, and haecceity of meaning and rejects ideas of context-free core meanings, defining stability as achieved stability.

More recently, Heritage and Stivers (2013, pp. 665) define a practice in the atomistic, reifying way as an empirical token that “(a) has a distinctive character, (b) has a specific location within a turn or sequence, and (c) is distinctive in its consequences for the nature or meaning of the action in which it is implemented” (drawing on Heritage, 2011, pp. 212). Practices are viewed as units that are positively identifiable and distinguishable-much like distinctive features as opposed to meaningful constituents in linguistics. Such units are sequentially placed in a particular location and adopt a specific role for the “nature or meaning” (Heritage and Stivers, 2013, pp. 665) or “function or meaning” (Sidnell, 2013, pp. 94) of the action they constitute. Comparably, for Schegloff (1993, pp. 121), practices are embodied in elements of conduct that ordinarily derive their “sense and import” for social action from their position and composition in the interactional event. For all three authors, practices have (or contribute to) a “meaning” (or sense) as well as a nature, function and import.

Turn-taking, for example, includes “core practices through which actions are designed, sequences are organized, and activities are accomplished in interaction” (Heritage, 1999, pp. 69). The interest of CA is to identify individual practices as units and their functions as well as sets of practices (Kitzinger, 2013, pp. 229). Because they are organized as interconnected totalities, sets of practice can accomplish institutional contexts that influence how particular interactional practices are understood by the actors (Mandelbaum, 2013, pp. 506). The reification of practices as independent units is a central difference between ethnomethodology and CA. This results in a further difference, which is that in CA texts it remains unclear who is considered to be the bearer of practices: individuals who insert practices as units into sequences and thus form utterances, or groups who share and understand them? Or are individuals and groups possibly produced by practices in the first place? While Garfinkel’s position is the latter, the CA literature is less clear in this regard.

Another theoretical consequence of the idea that practices are individual entities with an intrinsic meaning is that the scale of practices becomes relevant. Heritage and Stivers (2013, pp. 665) say that in constituting recognizable social actions, practices can operate at different levels ranging from prosody to word choice to turn organization and action construction. Larger and smaller practices are assumed, with the former sometimes encompassing the latter.

Therefore, Schegloff (as reported by Heritage, 1995, pp. 394, n.8, and Mandelbaum, 1990/1991, pp. 347) proposes a distinction between “practices of” and “practices in” ordinary conversation. “Practices of” conversation refer to the underlying organizational properties of social activities, i.e., the “mechanical features of talk” (Mandelbaum, 1990/1991, pp. 347) and the constitutive functions of practices. “Practices in” conversation, in comparison, refer to the activities, which participants perform in and through these mechanical features and for, with or on each other. “Most of these [latter] activities are vernacularly nameable-for example, questioning, complaining, challenging etc.-but not exclusively so. The term can also be employed characterize such activities as referring, listing or inviting recognition” (Heritage, 1995, pp. 394, n.8).

Comparably, Robinson (2007, pp. 68, n.2) distinguishes *practices* and *practices of action*. According to Sidnell’s interpretation, practices can constitute not only actions, but also practices of action, which are larger activities, but do not themselves yet belong to everyday action categories: “the former are conceptualized as constituting the latter. So, for instance, practices of turn design (i.e., interrogative format), lexical choice, intonation and gaze direction can all be combined in a single turn (…), in a context-sensitive way, to bring off the practice of action of selecting a next speaker” (Sidnell, 2013, pp. 98, n.2).

CA has reached an enormous level of granularity, identifying components that escape the attention of co-interactants because they are ephemeral and remain unnoticed. Some of the constitutive tasks of practices and building blocks of action can now only be named in specialized language that describes their function for the overall conversational organization (e.g., “other-initiated repair,” “projectability of turn-completion,” and “transitional overlap”), while others are still recognizable in members’ terms (“telling a joke,” “complaining,” and “interrupting”). The path from the meaning of practices to their function is sometimes short.

An interest in these “seen but unnoticed” (Garfinkel, 1967, pp. 36) background features of sociality is one of the motivations for using the concept of practice in CA. This interest is double-edged as, on the one hand, from the ethnomethodological perspective, it cannot be assumed that these background features are familiar for everybody. Rather, they are procedurally accomplished and thus respond to change, difference and disruption within the action itself. On the other hand, as Heritage and Stivers (2013, pp. 665) say, “the concept of practice describes characteristics of action that are independent of participants’ individual, personal or psychological characteristics.” CA understands practices as general or, as Maynard (2013, pp. 27) puts it, “generic and universal.” In recent years, CA increasingly used this type of universalist, anthropological language. Schegloff (2006, pp. 71), for example, declares a universal social “infrastructure” consisting of “half a dozen generic organizations of practice.” The reason of its existence is that “the organization of interaction needs to be-and is-robust enough, flexible enough, and sufficiently self-maintaining to sustain social order at family dinners and in coal mining pits, around the surgical operating table and on skid row, in New York City and Montenegro and Rossel Island, and so forth, in every nook and cranny where human life is to be found” (Schegloff, 2006, pp. 71). Sidnell (2007, pp. 241) explains that since “participation in conversation poses similar tasks and problems everywhere quite independently of the particular language used or the particular sociocultural setting in which the interaction takes place,” a “robust base of apparently generic interactional organization” reflecting “the specifically human ‘form of life’” is needed. Therefore, conversational turn-taking must be viewed as a function of the human species: “an adaptation to the contingencies of interaction between sighted, language-using bipeds” (Sidnell, 2001, pp. 1,265).

As we have seen, the term “practice” is used inconsistently in CA. Sometimes “practice” refers to a repertoire of tools that individuals use to perform actions, sometimes it relates to a (self-organized) interactional dynamic that draws members (not least morally) into producing a recognizable social phenomenon that appears external and objective. Sometimes “practices” appear like positively identifiable tokens that are used to build sequences (independent from them) to achieve local specifics of an interaction, sometimes they appear like tools used to accomplish the sequential organization of the interaction itself. Sometimes practices are universal, sometimes they are specific.

Returning to Garfinkel’s adoption of Gurwitsch’s concept of gestalt contexture discussed above, I suggest to complement essential criteria of practice in CA (specifically “context-sensitivity”) by “context-productivity.” To reflect the theoretical import of the concept of practice for CA more clearly I will draw on the example of the adjacency pair. Although the “core practices” in turn-taking are often located in the activities of individuals (e.g., “anticipatory completion,” “jump-start,” “rush-through” at transition relevance places), an impressive example of practices that powerfully produce co-participation, binding individuals together in a practice, are adjacency pairs and their “conditional relevance” (Schegloff, 2007, pp. 20). Examples are: greeting-greeting, question-answer, or offer-accept/decline. The components of adjacency pairs are typologized into first and second pair parts (what Gurwitsch calls “themes” and Garfinkel calls “details” or “indexical particulars”) that relate to the pair types which they compose (what Gurwitsch calls “thematic field” and Garfinkel calls “context”). A first pair part “projects a prospective relevance,” and makes relevant “a limited set of possible second pair parts, and thereby sets some of the terms by which a next turn will be understood” (Schegloff, 2007, pp. 16). Each item suggests a *next*. “Nextness” (Schegloff, 2007, pp. 14) along with “conditional relevance” is produced by the expectability of an adequate second pair part after a first pair part was provided. When a first pair part has been provided and a second pair part is being withheld, however, it becomes “noticeably absent” (Schegloff, 2007, pp. 20). The lack of an accomplished gestalt contexture (“good continuation”) entails considerable social consequences such as possible conflicts and reconfigurations of social relations. The “relationship of adjacency or ‘nextness’ between turns is central to the ways in which talk-in-interaction is organized and understood. Next turns are understood by co-participants to display their speaker’s understanding of the just-prior turn and to embody an action responsive to the just-prior turn so understood (unless the turn has been marked as addressing something other than just-prior turn)” (Schegloff, 2007, pp. 15). Thus, the procedural organization of intersubjectivity and social order here becomes dependent upon the practical, sequential organization of gestalt contextures by parties in a setting.

Schegloff has repeatedly pointed out that preceding utterances and actions sequentially form the context for ongoing utterances and actions. The adjacency pair example demonstrates this: it is not their intrinsic (context-free) meaning, but the sequential context that provides ongoing practices with meaning and makes them understandable-such as a brief delay after an invitation.

If we understand such conversational practices as the adjacency pair by reference to Gurwitsch as the continuous production of a contexture that conditions, or even compels, the provision of functionally indexed nexts by the co-interactant, we get a vision of conversational practice that unites context-sensitivity and context-productivity (and is maybe less interested in context-freeness). Since practices cannot be understood as context-free resources, but themselves, once past, form the context for their own continuation, adjacency pairs, as a practice, can be seen as a model for the joint formation of *in situ* self-organizing and self-constraining social objects that reflects the mutability, indeterminacy, and particularity of the social. The adjacency pair produces the context to which second pair parts are then sensitive.

This example shows a possible specification of the CA concept of practice: First, practices are not only sensitive to, but also productive of, the context, since they ongoingly establish it as gestalt contexture. Practices therefore can be seen as ongoingly producing those contexts as practical circumstances, which subsequent practices then continue context-sensitively by practical reasoning. Secondly, both meaning and function of practices are genuinely relational. The principle of gestalt contexture states that each *next* refers to a *before*, and there is no intrinsic meaning or function of the individual item. The idea that practices as resources have a general context-free core, which is then context-sensitively varied and adapted to local circumstances, can therefore be abandoned. Thirdly, practices are not individual but mutually complementary and continuous, accomplished by relationally emerging “members” in the double sense.

Returning to examples such as the adjacency pair could thus contribute to clarify the undecided position of CA with regard to the practical character of talk-in-interaction. They help avoiding anti-representationalist and routine-centered as well as structuralist models of practice that regard historically evolved commonalities as guarantees of social order and thus correspond to Aristotle’s original insight into the ongoing mutability, indeterminacy, and particularity of the social.

## Conclusion

As reconstructed in this text, Garfinkel presents an elaborate and consistent social-theoretical conceptualization of the terms “practice” and “practicality.” CA builds on this, even if it is theoretically less consistent, which is due to the tension between the notion of context freeness (with simultaneous context sensitivity) of the SSJ model and the idea of context productivity (with simultaneous context dependence) in the ethnomethodological model proper. While there are some differences, in particular the reifying use of the concept of practice (i.e., as “things,” ironically) and the assumption of the (shared) semantic stability of its units by CA scholars, these can be rethought with reference to the concept of “context-productivity.” The narrative sometimes put forward that CA has abandoned its theoretical foundations in ethnomethodology therefore does not entirely seem plausible. If one refers to Gurwitsch’s non-egological theory and not to Schütz’s egology, then even “practices” conceived as distinct units no longer appear as tools that actors voluntarily take from a toolbox and consciously use, but as situationally appropriate and promising components that constitute quasi-objective contexts of action. They produce and continue practical dynamics into which members of society are persistently drawn, as it is suggested by the notion of “machinery” prominent in ethnomethodology and CA.

From this perspective, both ethnomethodology and CA consistently adhere to the principle of the primordiality of practice and avoid that structural or intentionalist elements come back in through the backdoor, as is the case with some practice theories cited above. In this way, they do justice to the Aristotelian questions about the stability and continuity of the social in the face of the permanent mutability, fundamental indeterminacy and situational particularity of practical existence. Ethnomethodology and CA thus present a version of practice theory that avoids theoretical problems of the others, including the long-standing dualisms of agency versus structure or of routine versus rationality.

Ethnomethodology and CA do not subscribe to the idea that practice is primarily characterized by anti-representationalist immersion and absorbed coping. By adopting Gurwitsch’s critique on Heidegger, Garfinkel (1967, pp. 32–34) succeeds in maintaining the image of “serious,” “planful” actors as rationally reflecting upon and creatively operating in their situation. Members are viewed as constantly accomplishing in concert with others those features of social reality that they, as Merleau-Pontian “préjugé du monde,” attribute to the external, objective social world of Durkheimian things (Garfinkel, 1967, pp. 182). Other established approaches (Bourdieu, Giddens) do not completely exclude practical wisdom with their notions of “practical logic” and “practical consciousness,” but they place far more emphasis on routine and continuity than on reasoning and contingency than do Garfinkel and CA.

In this way, Garfinkel avoids explaining practice with the pre-existing sharedness of meanings or rules. While they are taken for granted in other practice theories, for Garfinkel they are in need of explanation. With his conception of the triangle of practical action, practical reasoning and practical circumstances, interactional practice appears as an ongoing mutual accomplishment of shared goals, means and processes, each reflected in the light of the other. Garfinkel argues that members constantly make social order discoverable for each other through the ongoing accomplishment of practices. This guarantees the continuity of the social. By assuming that members endlessly switch between theme and thematic field, no extrinsic factors are admitted theoretically as determining practice, or, sociologically speaking, no independent variables are accepted as valid explanations for social phenomena. CA could return to this position by adopting a third concept along with “context-freeness” and “context-sensitivity”: “context-productivity.” This allows for a more consistent formulation of practice that avoids the problems of other theories of practice.

Furthermore, in sociology, there is sometimes an image of ethnomethodology and CA as being overly detail-oriented and thus irrelevant to broader social analysis. That this judgment is based on an assumption that the social world is simply there rather than being constantly produced and continued in interaction, and that shared rules and meanings are predetermined rather than explained by concrete analyses of members’ practices, is shown by the ethnomethodological theoretical orientation. However, if one follows their concept of practice, it becomes clear why the emphasis on details is so important, because it is the details of the practices through which the supposedly objective world is produced in the first place.

For this reason, Garfinkel (2002, pp. 92, n.1) says that social order is *immortal*, for to speak of the immortality of ordinary society is “to speak of human jobs as of which local members, being in the midst of organizational *things,* know, of just *these* organizational things they are in the midst of, that it preceded them and will be there after they leave it.” Therefore, rather than to speak of “bundles of sayings and doings” or “webs of practices,” Garfinkel (2002, pp. 92, n.1) advises us to keep in mind that “the great recurrencies of ordinary society” present themselves in practical form, as “assemblages of haecceities,” which are co-constituted by reasoning members who are engaged in practical action within the particularity of situational circumstances.

## Author contributions

The author confirms being the sole contributor of this work and has approved it for publication.

## Footnotes

^1^As Garfinkel (2002, pp. 264-267) says, (formal sociological) theorizing is therefore in constant danger of “losing its phenomenon” as it moves away from both the observed practices and the practices used to obtain the data.

^2^While Heidegger sees practice as stabilizing human existence in general, it can also be understood in an ethnomethodological sense as stabilizing social order and social reality more specifically.

^3^There is a manuscript of 225 pages on Kotarbinski and ‘practical’ actions by Garfinkel in the Garfinkel Archive in Newburyport that still awaits evaluation.

^4^Although Harvey Sacks, who worked with Garfinkel for many years, was a prominent figure in the development of CA, I will not deal with his specific line of thought here in entirety. The reason for this is that Sacks only openly used the concept of practice in collaboration with Garfinkel, but not in the context of the emerging CA. Although some of his theoretical concepts could possibly be characterized as practice theoretical, for example the notion of “machinery” (*cf.* Meyer, 2018, pp. 68–69), his approach has also been portrayed as inspired by structuralist ideas (Schegloff, 1992, pp. xxi, xxxvi) or even as “primitive natural science” (Lynch and Bogen, 1994). Since Sacks rarely uses the terms “practice” or “practical,” it would require a larger hermeneutic exercise of characterizing Sacks as practice theoretical or not than is possible here. In general, as Schegloff (1992, pp. xi) emphasizes, Sacks was more interested in larger orders of conversational organization, than in particular practices, and in organizationally characterized forms of interactional work, than in individual outcomes.
